# Domestic cats (*Felis catus*) discriminate their names from other words

**DOI:** 10.1038/s41598-019-40616-4

**Published:** 2019-04-04

**Authors:** Atsuko Saito, Kazutaka Shinozuka, Yuki Ito, Toshikazu Hasegawa

**Affiliations:** 1 Department of Cognitive and Behavioral Science, Graduate School of Arts and Sciences, the University of Tokyo, 3-8-1 Komaba, Meguro-ku, Tokyo, Japan; 2 Department of Childhood Education, Musashino University, 1-1-20 Shinmachi, Nishitokyo-shi, Tokyo, Japan; 3 Department of Psychology, Faculty of Human Sciences, Sophia University, 7-1 Kioicho, Chiyoda-ku, Tokyo, Japan; 4 RIKEN Center for Brain Science, 2-1 Hirosawa, Wako, Saitama, Japan

**Keywords:** Human behaviour, Animal behaviour

## Abstract

Two of the most common nonhuman animals that interact with humans are domestic dogs (*Canis familiaris*) and cats (*Felis catus*). In contrast to dogs, the ability of domestic cats to communicate with humans has not been explored thoroughly. We used a habituation-dishabituation method to investigate whether domestic cats could discriminate human utterances, which consisted of cats’ own names, general nouns, and other cohabiting cats’ names. Cats from ordinary households and from a ‘cat café’ participated in the experiments. Among cats from ordinary households, cats habituated to the serial presentation of four different general nouns or four names of cohabiting cats showed a significant rebound in response to the subsequent presentation of their own names; these cats discriminated their own names from general nouns even when unfamiliar persons uttered them. These results indicate that cats are able to discriminate their own names from other words. There was no difference in discrimination of their own names from general nouns between cats from the cat café and household cats, but café cats did not discriminate their own names from other cohabiting cats’ names. We conclude that cats can discriminate the content of human utterances based on phonemic differences.

## Introduction

Domestic cats (*Felis catus*) and dogs (*Canis familiaris*) are the most popular companion animals; worldwide, over 600 million cats live with humans^1^, and in some countries their number equals or exceeds the number of dogs (e.g., Japan: dogs: 8,920,000, cats: 9,526,000)^2,3^. Cats started to cohabit with humans about 9,500 years ago^4^; their history of cohabitation with humans is shorter than that of dogs^5^, and they have been domesticated by natural selection, not by artificial selection^6–8^. Despite these differences in their process of domestication compared to that of dogs, cats too have developed behaviours related to communication with humans; for example, for human listeners, the vocalisations of domestic cats are more comfortable than those of African wild cats (*Felis silvestris lybica*)^9^. In addition, purring has different acoustical components during solicitation of foods than at other times, and humans perceive such solicitation purrs as more urgent and unpleasant than non-solicitation purrs^10^. These facts clearly indicate that domestic cats have developed the ability to communicate with humans and frequently do so; Bradshaw^8^ suggested that this inter-species communicative ability is descended from intra-species communicative ability.

Researchers have only recently begun to investigate cats’ ability to communicate with humans. Miklósi *et al*. showed that cats are able to use the human pointing gesture as a cue to find hidden food, similarly to dogs^11^. The researchers also suggested that cats do not gaze toward humans when they cannot access food, unlike dogs. However, a recent study revealed that cats show social referencing behaviour (gazing at human face) when exposed to a potentially frightening object, and to some extent cats changed their behaviour depending on the facial expression of their owner (positive or negative)^12^. Cats in food begging situations can also discriminate the attentional states of humans who look at and call to them^13^. In addition, Galvan and Vonk demonstrated that cats were modestly sensitive to their owner’s emotions^14^, and other research has indicated that cats’ behaviour is influenced by human mood^15,16^. Further, cats can discriminate their owner’s voice from a stranger’s^17^. This research evidence illustrates that domestic cats have the ability to recognize human gestural, facial, and vocal cues.

In contrast to cats, numerous research studies have shown the ability of domestic dogs to communicate with humans. Dogs are skilful at reading human communicative gestures, such as pointing (reviewed in Miklósi & Soproni^18^). Dogs can differentiate human attentional states^19–22^ and distinguish human smiling faces from blank expressions^23^. They are also capable of using some human emotional expressions to help them find hidden food and fetch objects^24,25^.

Although the majority of prior studies have focused on visual communication between humans and dogs^26^, some studies have investigated the dog’s ability to respond to human vocalisations. For example, the pitch of a human voice affects dog behaviour^27^: dogs obey high-pitched voices to a greater extent than low-pitched voices. Dogs can discriminate expressions of emotion with voice^28^, and obey a command with angry voice more slowly than with happy voice. Dogs trained to sit and come in response to tape-recorded commands change their performance when the phonemes of commands are changed^29^. Many dog owners believe their dogs understand about 30 utterances^30^. Extensively trained dogs are able to differentiate 200–1000 human words or labels^31,32^. The ability to understand human verbal utterances is also shown in other species, such as apes^33^, dolphins^34^, and parrots^35^; however, whether such an ability exists in domestic cats remains untested.

In the present study, we investigated the ability of domestic cats to discriminate human verbal utterances. Cats are sensitive to differences in human voice characteristics^17^. Some owners insist that their cats can recognize their own names and words related to food. Therefore, we can make the following hypothesis: cats can discriminate words uttered by humans from other words—especially their own names, because a cat’s name is a salient stimulus as it may be the human utterance most frequently heard by domestic cats (cats kept by humans) and may be associated with rewards, such as food, petting, and play.

We conducted experiments in cats’ homes, using a habituation-dishabituation method, as in our previous study^17^. In general, dogs’ ability to recognize human utterances are tested using command and retrieval tasks^31,36^. These tasks require pre-training, and the training of cats to perform on command would require a lot of effort and time. On the other hand, habituation-dishabituation method enabled us to measure cats’ natural reactions during a single visit, without extensive training. To test the hypothesis, we presented four different words serially as habituation stimuli, then presented the cats’ own names as test stimuli. If the cats were habituated to the other 4 words and dishabituated to their own names, a rebound response to the presentation of their own names would be observed, indicating the ability to discriminate their own names from other words.

We conducted four experiments to test the hypothesis. In Experiment 1, we investigated whether cats can discriminate their own names from general nouns with the same length and accents as their own names. If cats can discriminate their own names from other words by using phonetic characteristics other than length of or accent of stimuli, cats habituated to the other 4 words should show dishabituation when hearing their own names. The test cats were living either with no other cats or with a small number of other cats. In this experiment, although we equalized the familiarity of the nouns, the relative familiarity of names and other nouns was markedly different, that is, cats heard their own names more frequently than other nouns. Therefore, cats discriminated their own names depending both on phonetic characteristics and on familiarity. In Experiment 2, we investigated cats’ ability to discriminate their own names from other cats’ names, by using cats living with 4 or more other cats. It can be assumed that the test cats were exposed to the other cats’ names as well as their own names; stimuli were prepared using cohabiting cats’ names. Then, in Experiment 3, we examined effects of multiple-cat living environments on discrimination of general nouns and cats’ own names, similar to Experiment 1. In Experiments 2 and 3, we tested cats both from ordinary households and from a ‘cat café’, a business establishment where visitors can freely interact with cats. In Experiments 1 to 3, stimuli used cats’ owners’ own voices, because they exhibit a marked response to their owner’s voice^17^. However, this leaves open the possibility that cats can discriminate their own names only when their owners utter them. Thus, in Experiment 4, we tested whether cats can discriminate their own names from general nouns even when unfamiliar persons utter them; if they showed discrimination ability in this experiment, we would take them to recognize their own names based on common phonetic characteristics in human verbal utterances.

## Results

### Behaviour score

**Figure 1:**
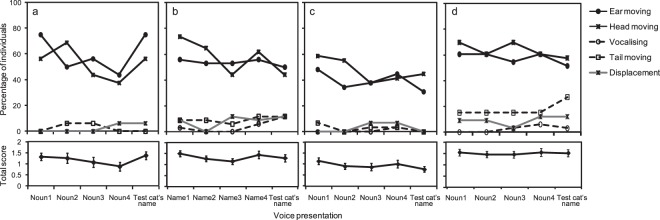
Response style to vocal stimuli in overall cats. Upper panels: Behaviour observed in response to voice stimuli and the percentage of cats that expressed each behaviour in (**a**) Experiment 1, (**b**) Experiment 2, (**c**) Experiment 3, and (**d**) Experiment 4. Black solid lines indicate orienting response. Black dashed lines indicate communicative response. Gray solid lines indicate displacement. Lower panels: Mean total behavioural scores for all cats in (**a**) Experiment 1, (**b**) Experiment 2, (**c**) Experiment 3, and (**d**) Experiment 4. Error bars indicate SEs.

The upper panels of Fig. 1 summarise the cats’ responses to the stimuli, as scored by the experimenter. Through all the experiments, more than half of the cats responded to voice stimuli by moving their ears and heads; fewer than 10% of the cats demonstrated vocalisation, tail movement, and displacement. This trend did not differ contingent on whether stimuli were nouns, other cats’ names, or tested cats’ own names. Fisher’s exact test revealed that number of cats which showed orienting response (moving ear and/or moving head) were significantly higher than which showed communicative response (vocalization and/or tail movement) in all trials from Experiment 1 to 4 (Supplementary Table S1).

The total scores (Fig. 1 lower panels) were moderately correlated with the average response magnitude evaluated by the raters, as shown in the next section (Spearman’s rank correlation, *ρ* = 0.70, *P* < 0.001; *ρ* = 0.61, *P* < 0.001; *ρ* = 0.64, *P* < 0.001, *ρ* = 0.60, *P* < 0.001 for Experiments 1, 2, 3, and 4, respectively). Thus, the raters’ evaluations of the response magnitudes might have partly depended on the number of simultaneously occurring responses by the cats.

### Response magnitude

**Figure 2:**
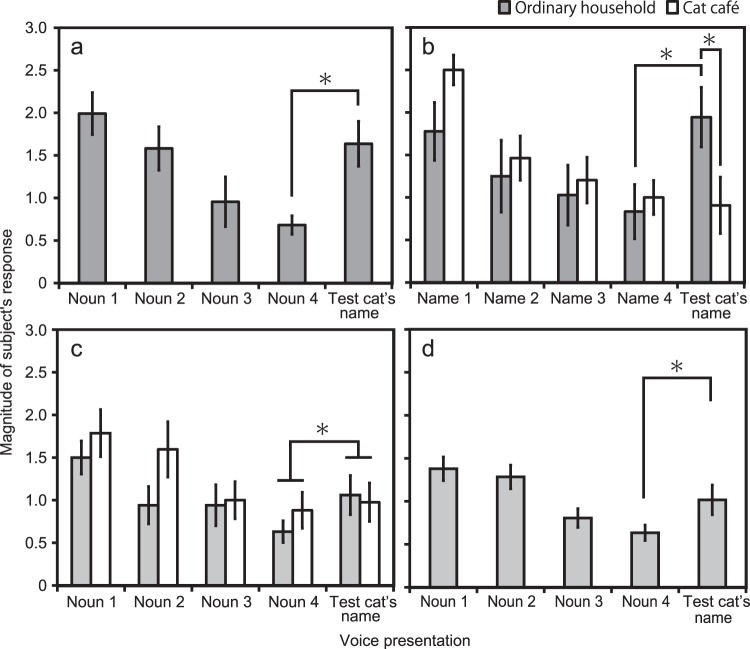
Mean magnitude of responses to each voice in habituated cats in (**a**) Experiment 1, (**b**) Experiment 2, (**c**) Experiment 3, and (**d**) Experiment 4. Error bars indicate SEs. Asterisks indicate significant differences (*P* < 0.05).

In Experiment 1, the raters’ evaluations revealed that eleven out of the 16 test cats decreased their average response magnitude from noun 1 to noun 4. These cats were considered to have successfully habituated to the general nouns vocalised by the owners. Then, nine out of the eleven habituated cats increased their response magnitude from noun 4 to their own name. Group-level analysis using a generalized linear mixed model (GLMM) revealed a significant effect of stimulus category (*F*(1,10) = 11.18, *P* = 0.007), indicating eleven habituated cats significantly increased in response magnitude from noun 4 to their own name (*t*(10) = −3.34, *P* = 0.007, Fig. 2a). Thus, habituated cats dishabituated when they heard their own names.

In Experiment 2, 15 out of the 34 test cats decreased average response magnitude from name 1 to name 4, and were considered to have successfully habituated to stimuli consisting of the names of other cohabiting cats. The ratio of successfully habituated cats was different between the ordinary households and the cat café (ordinary households: 6 out of 24, cat café: 9 out of 10; χ^2^ = 9.60, *df* = 1, *P* = 0.002). Although the ratio of successfully habituated cats from ordinary households is very low, we analysed the data from these six cats because of methodological restriction. We added housing environment (ordinary households or cat café) as a fixed effect for group-level analysis. GLMM revealed a significant effect of interaction (stimulus category * environment; *F*(1,13) = 8.26, *P* = 0.013). All six habituated cats from ordinary households increased their response magnitudes from name 4 to their own names. Post-hoc analysis revealed that response magnitudes to stimuli for cats’ own names were significantly higher than those for name 4 in these six habituated cats (*t*(13) = −3.43, *P* = 0.005, Fig. 2b). In contrast, only three out of nine habituated cats from the cat café increased their response magnitudes from name 4 to their own name. The response magnitudes to cats’ own names did not differ from those to name 4 in these nine habituated cats (*t*(13) = 0.35, *P* = 0.732, Fig. 2b). Significantly higher response was also seen in household cats compared to café cats in response to own name (*t* (20.24) = −2.39, *P* = 0.027), but not in response to noun 4 (*t* (20.24) = 0.38, *P* = 0.705, Fig. 2b).

In Experiment 3, following the results of Experiment 2, we again included the environment as a fixed effect. Fourteen out of the 20 household cats decreased average response magnitude from noun 1 to noun 4. Seven out of nine café cats decreased average response magnitude from noun 1 to noun 4. These cats were considered to have successfully habituated to the stimuli consisting of spoken nouns. In contrast to Experiment 2, interaction of stimulus category * environment was not significant (*F* (1,19) = 1.52, *P* = 0.233). A final model only included the effect of stimulus category (*F* (1,20) = 6.05, *P* = 0.023). Thirteen out of the 21 habituated cats increased their response magnitude from noun 4 to their own name. The response magnitude to cats’ own names was significantly higher from that to noun 4 in these 21 habituated cats (*t*(20) = −2.46, *P* = 0.023, Fig. 2c). Thus, these habituated cats dishabituated when they heard their own names.

In Experiment 4, the raters’ evaluations revealed that 20 out of the 33 test cats decreased their average response magnitude from noun 1 to noun 4; these cats were considered to have successfully habituated to the general nouns vocalised by unfamiliar persons. Then, 13 out of the 20 habituated cats increased their response magnitude from noun 4 to their own name. Group-level analysis revealed a significant effect of stimulus category in twenty habituated cats (*F*(1,19) = 4.41, *P* = 0.049), who dishabituated significantly when they heard their own name uttered by an unfamiliar person as compared to noun 4 (*t*(19) = −2.10, *P* = 0.049, Fig. 2d).

We also analysed habituated cats’ sum of behaviour score (total score) to test whether number of responses simultaneously elicited in response to a vocal stimulus increased from trial 4 (noun or other cat’s name) to trial 5 (test cat’s name). However, unlike response magnitude, significant increase in the total score was not observed except for Experiment 2 (Supplementary Fig. S1). This result suggests that qualitative analysis of behaviour with present/absent manner is less sensitive to detect dishabituation. It is confirmed that effectiveness of quantitative analysis with the response magnitude coded by blind raters.

## Discussion

In Experiments 1, 3, and 4, cats that habituated to general nouns with the same length and accent as their own names dishabituated to their own names. This was true both when their owner’s voice was presented (Experiments 1 and 3) and when the unfamiliar person’s voice was presented (Experiment 4), in spite of the fact that cats distinguish owners’ voices from unfamiliar persons’ voices^17^. These results show that cats can identify their own names from other words that consisted of the same number of mora but with different phonemes when they are uttered both by familiar person and by unfamiliar person. The results of Experiment 2 suggest that cats from ordinary households discriminate their own names from those of cohabiting cats but that cats from a cat café may not. From the results of all experiments, it thus appears that at least cats living in ordinary households can distinguish their own names from general words and names of other cats. This is the first experimental evidence showing cats’ ability to understand human verbal utterances.

How can we explain this ability and behaviour on the part of the cats? Their own names must be one of the most-heard human utterances by cats. If they have no meaning, frequently experienced stimuli should be habituated and not elicit reaction from cats. However, the results of our experiment were to the contrary; thus, the association between hearing their names and receiving rewards or punishments might affect the behaviour of cats. This implies that cats’ names can be associated with rewards, such as food, petting, and play, or with punishments, such as taking them to a veterinary clinic or to a bath. Sometimes, owners who keep multiple cats will call all of their cats’ names at the same time. In that situation, a cat may associate both its own name and those of cohabiting cats with reward. These situations could explain the results of Experiment 2: the ratio of ordinary household cats that successfully habituated to names of other cohabiting cats was very low (6 out of 24). There is a possibility that cats housed with other multiple cats may associate other cats’ names with rewarding or unpleasant events. However, in some situations, for example, when the owner wishes to take it to a veterinary clinic, or to pet a cat, they may call only one cat’s name. Taking them to clinic should be a stressor. Petting could be rewarding to the cat^37^, although depending on the cats’ personality, it could also be a stressor^38^. These situations would facilitate a cat’s learning to discriminate its own name from those of other cats.

If cats associate their own name with rewards or stressors, it is reasonable to think that they react to their name. In these experiments, cats responded to owner vocalisation not with communicative behaviour (vocalisation and tail moving)^39^ but just with orienting behaviour (ear moving and head moving)^40^. This tendency replicated that reported in our previous study^17^. This may be caused by the difference between the situation where we conducted the experiments and the natural situation. In normal reward or stress situations, name calling by owners may elicit more dynamic, or communicative reaction from cats.

Next, we consider the results from the cat café. The café cats did not discriminate their own names from the names of cohabiting cats, though their performance in the discrimination of their own names from general nouns did not differ from that of ordinary household cats. The social environment may explain this difference in results. Many different humans visit cat cafés, and since the cats’ names are listed in cafés, visitors can call the names of the cats. However, the way names are called may vary by visitor (e.g., intonation may vary); such a condition may hinder cats in discriminating their name from those of other cats. Or, café cats may hear their name mentioned along with other cat names frequently without accurate reward discrimination by visitors. For example, if a visitor calls cat A, but cat B approaches to the visitor and cat B gets petting and treats instead of cat A. These situations would make name discrimination less relevant for these cats. Additionally, the number of cohabiting cats may have affected the results. Usually, the number of cats in a cat café is greater than the number in an ordinary household. Further, because we conducted the experiment in only one cat café, we cannot assure their generalisability or reach a definitive conclusion.

Nevertheless, this study has demonstrated that cats can discriminate human utterances based on phonemic differences. Although such discrimination is acquired without explicit discrimination training, instead emerging from the patterns of natural daily communication between humans and cats, we may utilise this ability positively for cats’ quality of life. For example, perhaps we can get cats to learn that dangerous objects or places are referred to by specific utterances. This work has shed new light on the ability of cats to communicate with humans; further clarifying cats’ abilities with respect to cat–human communication will potentially enhance the welfare of both humans and cats.

## Methods

### Subjects

In Experiment 1, the participants were 16 domestic cats (8 males and 8 females; age range: 1–11 years, mean age: 3.69 years, *SD* = 3.01) living with 11 families (three male and eight female owners), each of whom lived with 2 or fewer other cats. By breed, there were 12 mongrels, two Scottish Folds, an American Shorthair, and a Himalayan. Fifteen of the cats had begun to live with their owner within one year of birth, and one cat when it was 5 years old. Fifteen of the cats were neutered (one female was not).

In Experiment 2, 34 domestic cats (16 males and 18 females) each of which was living with 4 or more other cats, participated. Twenty-four cats were owned by four families and the remaining 10 were part of a ‘cat café’, a business establishment where visitors can freely interact with cats. The cats had six female owners (two owners were members of the same household). Of the 34 cats, there were 24 mongrels, three LaPerms, a Devon Rex, a Somali, a Scottish Fold, an American Curl, a LaPerm Shorthair, a Tonkinese, and a Munchkin. Their ages ranged from 0.5 to 10 years (mean age: 5.51 years, *SD* = 2.95), and the ages when they began to live with their owners ranged from birth to 36 months after birth. All cats were neutered.

In Experiment 3, participants were 29 domestic cats (16 males and 13 females) living with 4 or more other cats. They were kept by three families and one cat café, which had four female owners; of the 29 cats, 9 were from the cat café. Breeds were 21 mongrels, three LaPerms, a Scottish Fold, an American Curl, a LaPerm Shorthair, a Tonkinese, and a Munchkin. Their ages ranged from 1 to 11 years (mean age: 6.48 years, *SD* = 3.29). The ages when they began to live with their owners ranged from birth to 36 months after birth. All cats were neutered. Of these 29 cats, 26 cats participated in Experiment 2. Interval between Experiment 2 and 3 was at least 2 weeks.

In Experiment 4, participants were 33 domestic cats (14 males and 19 females) living with from 0 to 5 other cats. Of them, 30 cats were kept in 21 families (2 male and 19 female owners) and 3 cats were kept in university laboratories. Of the 33 cats, 24 were mongrels, two LaPerms, two American Shorthair, a Scottish Fold, a Himalayan, a Russian Blue, a Norwegian Forest Cat, and a Bengal. Their ages ranged from 1 to 17 years (mean age: 6.48 years, *SD* = 4.14), and the ages when they began to live with their owners ranged from one month to 36 months after birth. All cats were neutered, excepting one female. Of these 33 cats, 3 had participated in Experiment 1 and 5 had participated in Experiments 2 and 3. Experiment 4 was conducted about 3 years after Experiment 3. In all experiments, all cats were indoor only except one, and cats were not subjected to food deprivation during the study period. Detailed information is presented in the electronic Supplementary Material (Tables S2–S5).

### Apparatus and Stimuli

Before the experiments began, for each cat, five sound stimuli consisting of human voice were recorded. One stimulus consisted of a human calling the cat’s name. The other four stimuli consisted of a human vocalising four different general nouns (Experiments 1, 3, and 4) or four names of other cats living with the test cats (Experiment 2). For Experiments 1, 2, and 3, the stimuli were recorded by the owners of the tested cats. For Experiment 4, the stimuli were recorded by two women unfamiliar to the tested cats. Each owner was instructed to vocalise the cat’s names as he/she normally would; if the owner usually called the cat by a nickname instead of its real name, the nickname was used. In Experiments 1, 3, and 4, four different general Japanese nouns were selected from the list of Matsumoto^41^; all nouns had the same level of familiarity and were emotionally neutral. The numbers of moras and accents in the nouns were the same as in the cat’s name. Speakers were instructed to vocalise the nouns with the same intonation and manner as they vocalised the cats’ names. In Experiment 2, four of the other cohabiting cats’ names were recorded similarly to the test cats’ names. The orders of presentation of general nouns and cohabiting cats’ names were pseudo-randomized.

We recorded the vocalisations with a handheld digital audio recorder (ZOOM H2 Handy Recorder) in WAV format; the sampling rate was 44100 Hz with 16-bit quantisation. The sound stimuli were adjusted to the same volume level using sound editing software (Adobe Soundbooth CS4 or Adobe Audition CS6). During the experiment, the handheld recorder was used to present the stimuli through a speaker (Sony SRS-Z100), which was hidden from the test cat. The distance between the test cat and the speaker was about 3 m, and the volume of the voices was approximately 65 dB at 3 m from the speaker. A video camera (Sanyo DNX-CA9 or Panasonic HX-WA20) placed in front of the test cats recorded their reactions during the playback of the stimuli.

For Experiment 1, 3, and 4, the discriminant analysis was performed to confirm that there was no implicit difference in acoustic characteristics between noun and name stimuli. Vocal stimuli for cats which showed dishabituation (habituated cats with increasing response magnitude from noun 4 to own name: *N* = 9, 13, and 13 in Experiment 1, 3, and 4, respectively) were selected for analysis. Six acoustic parameters were extracted from each vocal stimulus by using Praat 6.0.43 software: total duration (sec), mean pitch (Hz), f1 (Hz), f2 (Hz), f3 (Hz), and mean intensity (dB). Then the discriminant analysis was applied with IBM SPSS Statistics 21. Above acoustic parameters were set as independent variables, and type of stimulus (noun or name) was set as a group. As a result of the analysis, high values of Wilks lambda were obtained (Experiment 1, Wilks lambda = 0.930, χ^2^ = 2.884, *df* = 6, *P* = 0.823; Experiment 3, Wilks lambda = 0.821, χ^2^ = 11.866, *df* = 6, *P* = 0.065; Experiment 4, Wilks lambda = 0.979, χ^2^ = 1.294, *df* = 6, *P* = 0.972), indicating that it was difficult to discriminate between noun and name stimuli by using implicit acoustical characteristics as a cue.

### Procedure

Experiments 1, 2, and 3 were conducted from December 2012 to November 2013; Experiment 4 was conducted from September 2016 to April 2017. All experiments were held in each owner’s home or in the cat café, wherever the particular cats lived. The experimenter waited until cats were calm before beginning the experiment. During the experiment, the owners were out of their cat’s sight. We used a habituation-dishabituation procedure in which prepared stimuli were played serially with a 15-s inter-stimulus interval (ISI); the order of presentation was word 1, word 2, word 3, word 4, and test cat’s name. The number of habituation stimuli and the ISI were improved versions of those used in a previous study^17^. Cats’ responses to the stimuli were expected to decrease during the presentation of words 1 through 4 due to habituation; then, if the cats could discriminate their own names from the other words, responses were expected to increase again when their own names were presented, due to dishabituation. The experiment lasted around 1.5 minutes. During presentation, the test cat was not actively isolated from cohabiting cats, to keep the test cat’s behaviour natural. There was no need for any interruption in the experimental sessions due to cohabiting cats’ behaviour.

All procedures related to animal care and experimentation in our research adhered to the ‘Guidelines for the treatment of animals in behavioural research and teaching’ as published by the Association for the Study of Animal Behaviour in *Animal Behaviour* 71, 245–253 (2006) and to the ethical guidelines of the University of Tokyo. The study was approved by the Animal Experiments Committee of the Graduate School of Arts and Sciences of the University of Tokyo and by the Animal Experiments Committee of Musashino University.

### Behavioural analysis

Video-recordings of cats’ responses were trimmed to show from 5 s before stimulus onset to 10 s after stimulus offset, using Adobe Premiere CS6. Vocalisation of the words and cats’ names in the clips was masked by pure tones to facilitate blind evaluation of the clips. In total, 80, 170, 145, and 165 clips were created for Experiments 1, 2, 3, and 4, respectively.

We conducted two kinds of analyses to investigate the cats’ response styles and magnitudes, as in our previous study^17^. The first analysis describes response style. One of the experimenters (KS) observed the clips of each cat in random order and classified the cat’s responses to the stimuli into five categories: ear moving, head moving, vocalising, tail moving, and displacement; each category is described in Table 1. These categories cover orienting responses (ear moving and head moving)^40^ and communicative responses (vocalising and tail moving)^39^. Each category was scored separately as 0 (absent) or 1 (present) for each clip, to determine the proportion of cats showing each response in each presentation trial. Then, the summed score was calculated as the total score for each clip, to enable examination of the correlation between the numbers of categories occurring simultaneously and response magnitude rated by blind raters (described in the next section). To check for reliability, the other experimenter (AS) observed a random selection of one-fourth of the clips and scored the cats’ behaviours. The indices of concordance were 0.75 for ear moving, 0.81 for head moving, 0.99 for vocalising, 0.97 for tail moving, and 0.99 for displacement (κ = 0.76, *P* < 0.001 for overall observation).

**Table 1:** Descriptions of categories for behavioural scores.

Category	Description
Ear moving	Any change in ear(s) angle from ear root
Head moving	Any change in head angle at the neck
Vocalising	Any vocalisation
Tail moving	Any movement of tail between its root and tip
Displacement	More than one step of displacement of both hind paws in any direction

The second analysis was conducted to examine response magnitude. Raters who were blind to the stimuli and their presentation order scored each cat’s responses in the clips, which were presented in random order within each test cat. In Experiment 1, there were ten blind raters (6 men and 4 women; mean age = 21.7 years), whereas in Experiment 2 and 3 there were six blind raters (all women; mean age = 27.5 years), and in Experiment 4, nine blind raters (one man and 8 women; mean age = 22.9 years). The raters were instructed to compare each cat’s behaviours before and after the presentation of each stimulus and rate the magnitude of the cat’s responses to the stimuli from 0 (no response) to 3 (marked response). Kendall’s coefficient of concordance showed significant, moderate concordance among the raters (*W* = 0.73, *df* = 79, *P* < 0.001; *W* = 0.73, *df* = 169, *P* < 0.001; *W* = 0.65, *df* = 144, *P* < 0.001, *W* = 0.55, *df* = 164, *P* < 0.001 for Experiments 1, 2 3, and 4, respectively).

Mean response magnitude was calculated for each video clip and used for subsequent analysis. GLMM was applied using the lme4 package version 1.1–13 on R software version 3.4.1. Stimulus category (Experiment 1; noun 4 v. own name, Experiment 2; other cat’s name 4 v. own name, Experiment 3; noun 4 v. own name, Experiment 4; noun 4 v. own name) was set as a fixed effect. Environment (ordinary households v. cat café) and interaction of stimulus category * environment were also set as fixed effects for Experiments 2 and 3. Subjects were set as a random effect. Gaussian distribution with identity link function was specified for lmer function. Then, post-hoc analysis was conducted using the step function in the lmerTest package version 2.0–33; the step function reduced non-significant fixed effects and determined a final model. The random effect (subjects) was manually kept regardless of significance, to control pseudo-replication.

## Supplementary information

Supplementary tables and figure

Dataset

## Data Availability

The data supporting this article are included in Supplementary Electronic Information.
