# Author Correction: Domestic cats (*Felis catus*) discriminate their names from other words

**DOI:** 10.1038/s41598-019-46257-x

**Published:** 2019-09-10

**Authors:** Atsuko Saito, Kazutaka Shinozuka, Yuki Ito, Toshikazu Hasegawa

**Affiliations:** 1 Department of Cognitive and Behavioral Science, Graduate School of Arts and Sciences, the University of Tokyo, 3-8-1 Komaba, Meguro-ku, Tokyo, Japan; 2 Department of Childhood Education, Musashino University, 1-1-20 Shinmachi, Nishitokyo-shi, Tokyo, Japan; 3 Department of Psychology, Faculty of Human Sciences, Sophia University, 7-1 Kioicho, Chiyoda-ku, Tokyo, Japan; 4 RIKEN Center for Brain Science, 2-1 Hirosawa, Wako, Saitama, Japan

Correction to: *Scientific Reports* 10.1038/s41598-019-40616-4, published online 04 April 2019

This Article contains typographical errors in the Methods section, under the subheading ‘Subjects’,

“Their ages ranged from 1 to 11 years (mean age: 6.48 years, SD = 3.29).”

should read:

“Their ages ranged from 1 to 12 years (mean age: 6.48 years, SD = 3.29).”

In addition,

“Of them, 30 cats were kept in 21 families (2 male and 19 female owners) and 3 cats were kept in university laboratories.”

should read:

“Of them, 30 cats were kept in 19 families (1 male and 18 female owners) and 3 cats were kept in university laboratories (1 male and 1 female owners).”

Furthermore,

“Of these 33 cats, 3 had participated in Experiment 1 and 5 had participated in Experiments 2 and 3.”

should read:

“Of these 33 cats, 4 had participated in Experiment 1 and 5 had participated in Experiments 2 and 3.”

Finally, in the Supplementary Tables and Figure file, in Table S5 the ages of subjects Ngboi and Nnn were reversed and should read ‘2’ and ‘7’ respectively.

