# Supplementary material for: Domestic cats (Felis catus) discriminate their names from other words
Source: Sci Rep. 2019 Apr 4;9:5394. doi: 10.1038/s41598-019-40616-4 (PMC6449508; doi:10.1038/s41598-019-40616-4)
Supplement: Supplementary file 1 — Supplementary tables and figure [file 41598_2019_40616_MOESM1_ESM.pdf]

Domestic cats (*Felis catus*) discriminate their names from other words

Atsuko Saito*†[1,2,3], Kazutaka Shinozuka†[4], Yuki Ito[1], Toshikazu Hasegawa[1]

[1]Department of Cognitive and Behavioral Science, Graduate School of Arts and Sciences, the University of Tokyo, 3-8-1 Komaba, Meguro-ku, Tokyo, Japan

[2] Department of Childhood Education, Musashino University, 1-1-20 Shinmachi, Nishitokyo-shi, Tokyo, Japan

[3] Department of Psychology, Faculty of Human Sciences, Sophia University, 7-1 Kioicho, Chiyoda-ku, Tokyo, Japan

[4]RIKEN Center for Brain Science, 2-1 Hirosawa, Wako, Saitama, Japan

*Corresponding author

†These authors contributed equally.

Table S1. Statistical analysis for behaviour score. Results of Fisher's exact test for number of cats which showed orienting response (ear moving and/or head moving) and communicative response (vocalising and/or tail moving) in each voice presentation were shown. *P* values were adjusted with Bonferroni correction for each experiment.

Experiment	Voice presentation	Odds ratio	95% confidence interval		Adjusted *P* value	
1	Noun 1	0.000	0.000	0.084	0.000	***
	Noun 2	0.026	0.000	0.261	0.001	***
	Noun 3	0.045	0.001	0.429	0.010	**
	Noun 4	0.000	0.000	0.314	0.004	**
	Test cat's name	0.000	0.000	0.084	0.000	***
2	Name 1	0.019	0.003	0.089	0.000	***
	Name 2	0.019	0.003	0.089	0.000	***
	Name 3	0.024	0.002	0.125	0.000	***
	Name 4	0.040	0.008	0.155	0.000	***
	Test cat's name	0.165	0.046	0.529	0.006	**
3	Noun 1	0.026	0.002	0.142	0.000	***
	Noun 2	0.000	0.000	0.084	0.000	***
	Noun 3	0.031	0.001	0.238	0.000	***
	Noun 4	0.036	0.003	0.191	0.000	***
	Test cat's name	0.000	0.000	0.110	0.000	***
4	Noun 1	0.051	0.011	0.195	0.000	***
	Noun 2	0.043	0.009	0.168	0.000	***
	Noun 3	0.043	0.009	0.168	0.000	***
	Noun 4	0.076	0.019	0.268	0.000	***
	Test cat's name	0.082	0.019	0.293	0.000	***

Table S2. Information on cats and their owners in Experiment 1

Cats				Owners	
**ID**	**Sex**	**Age (years)**	**Breed**	**ID**	**Gender**
Cr	Female	2	mongrel	RS	Female
Ekcn[1]	Female	1	Himalayan	TM	Female
Icg	Female	1	mongrel	YM	Male
Knt	Male	4	mongrel	MN	Male
Kro	Male	4	mongrel	KB	Female
Krr	Male	6	mongrel	AR	Female
Kucn	Male	10	mongrel	AK	Female
Mm	Female	2	mongrel	YM	Male
Nk	Female	1	American Short-hair	TM	Female
Okr	Male	5	mongrel	AS	Female
Rnk	Female	11	mongrel	YJ	Female
Sco	Male	4	mongrel	SZ	Male
Sm	Female	4	mongrel	KB	Female
Sr	Male	1	Scottish Fold	AT	Female
Te	Female	1	mongrel	RS	Female
Um	Male	2	Scottish Fold	AT	Female

[1]This cat was not neutered.

Table S3. Information on cats and their owners in Experiment 2

Cats				Owners	
**ID**	**Sex**	**Age (years)**	**Breed**	**ID**	**Gender**
Ann	Female	8	mongrel	TS	Female
Anr	Male	10	mongrel	TS	Female
Arcn	Female	2	mongrel	YK	Female
Bicn	Female	2	mongrel	TS	Female
Br	Female	10	mongrel	YA	Female
Brcn	Female	0.5	Devon Rex	MO	Female
Ck	Female	8	mongrel	YA	Female
Cocn[2]	Male	4	mongrel	KY	Female
Ct	Male	7	mongrel	YK	Female
Era[2]	Female	3	mongrel	KY	Female
Fucn	Male	10	mongrel	YA	Female
Hy	Male	10	mongrel	YA	Female
Jndu	Male	1	LaPerm	MO	Female
Knk[2]	Male	6	Somali	KY	Female
Kr	Male	5	mongrel	TS	Female
Mc[2]	Male	6	Scottish Fold	KY	Female
Mg	Female	5	mongrel	YA	Female
Mkn[2]	Female	5	American Curl	KY	Female
Mkzo	Male	2	LaPerm Shorthair	MO	Female
Nkkn	Male	6	mongrel	TS	Female
Nn	Female	9	mongrel	YK	Female
Pugi[2]	Male	5	Tonkinese	KY	Female
Rb[2]	Female	6	Munchkin	KY	Female
Rck	Female	2	LaPerm	MO	Female
Rk	Female	4	mongrel	TA	Female
Rncn	Female	5	mongrel	YK	Female
Ro	Male	10	mongrel	TA	Female
Sbcn	Female	5	LaPerm	MO	Female
Sk	Female	2	mongrel	YK	Female
Skr[2]	Female	3	mongrel	KY	Female
Srtm[2]	Male	3	mongrel	KY	Female
Tm	Male	9	mongrel	TA	Female
Uucn	Male	10	mongrel	TA	Female
Yz[2]	Female	4	mongrel	KY	Female

[2]These cats were from a "cat café".

Table S4. Information on cats and their owners in Experiment 3

Cats				Owners	
**ID**	**Sex**	**Age (years)**	**Breed**	**ID**	**Gender**
Ab[3]	Male	9	mongrel	TA	Female
Ann	Female	9	mongrel	TS	Female
Anr	Male	11	mongrel	TS	Female
Bicn	Female	3	mongrel	TS	Female
Br	Female	11	mongrel	TA	Female
Cocn[2]	Male	4	mongrel	KY	Female
Era[2]	Female	3	mongrel	KY	Female
Fucn	Male	11	mongrel	TA	Female
Hy	Male	11	mongrel	TA	Female
Jg[3]	Male	5	mongrel	TA	Female
Jndu	Male	1	LaPerm	MO	Female
Kr	Male	6	mongrel	TS	Female
Mc[2]	Male	6	Scottish Fold	KY	Female
Mg	Female	6	mongrel	TA	Female
Mkn[2]	Female	5	American Curl	KY	Female
Mkzo	Male	2	LaPerm Shorthair	MO	Female
Mnr[3]	Female	12	mongrel	TA	Female
Nkkn	Male	7	mongrel	TS	Female
Pugi[2]	Male	5	Tonkinese	KY	Female
Rb[2]	Female	6	Munchkin	KY	Female
Rck	Female	3	LaPerm	MO	Female
Rk	Female	5	mongrel	TA	Female
Ro	Male	11	mongrel	TA	Female
Sbcn	Female	5	LaPerm	MO	Female
Skr[2]	Female	3	mongrel	KY	Female
Srtm[2]	Male	3	mongrel	KY	Female
Tm	Male	10	mongrel	TA	Female
Uucn	Male	11	mongrel	TA	Female
Yz[2]	Female	4	mongrel	KY	Female

[2]These cats were from a "cat café".

[3]These cats did not participate in Experiment 2

Table S5. Information on cats and their owners in Experiment 4

Cats				Owners	
**ID**	**Sex**	**Age (years)**	**Breed**	**ID**	**Gender**
Ann[5]	Female	12	mongrel	TS	Female
Bicn[5]	Female	6	mongrel	TS	Female
Ci	Female	6	mongrel	TS	Female
Dnk	Male	8	mongrel	IN	Female
Dr	Male	5	mongrel	HU	Male
Ekc(n)[1, 4]	Female	5	Himalayan	TM	Female
Gr	Male	5	Bengal	JO	Female
Htr	Female	5	mongrel	MI	Female
Krm	Female	11	American Short-hair	SN	Female
Kt	Male	5	mongrel	IT	Female
Ktr	Male	5	mongrel	TN	Male
Micn	Male	17	mongrel	JO	Female
Mk	Male	5	mongrel	YY	Female
Mm	Female	5	mongrel	IT	Female
Mq	Male	2	mongrel	HU	Male
Mtb	Female	13	Scottish Fold	YS	Female
Ngboi	Male	7	Norwegian Forest Cat	MM	Female
Nk[4]	Female	5	American Short-hair	TM	Female
Nkkn[5]	Male	10	mongrel	TS	Female
Nnn	Female	2	Russian Blue	MM	Female
Okr[4]	Male	9	mongrel	AS	Female
Rc(k)[5]	Female	5	LaPerm	MO	Female
Rn(k)[4]	Female	15	mongrel	YJ	Female
Ron	Male	1	mongrel	NK	Female
Rq	Male	2	mongrel	KU	Female
Sbcn[5]	Female	8	LaPerm	MO	Female
Sc	Female	6	mongrel	AA	Female
Suk	Female	3	mongrel	CA	Female
Tb	Female	14	mongrel	MI	Female
Tncn	Female	2	mongrel	MOI	Female
To	Female	1	mongrel	KU	Female
Uocn	Female	1	mongrel	MOI	Female
Uzr	Male	8	mongrel	ASK	Female

[1]This cat was not neutered.

[4]These cats participated in Experiment 1.

[5]These cats participated in Experiments 2 and 3.

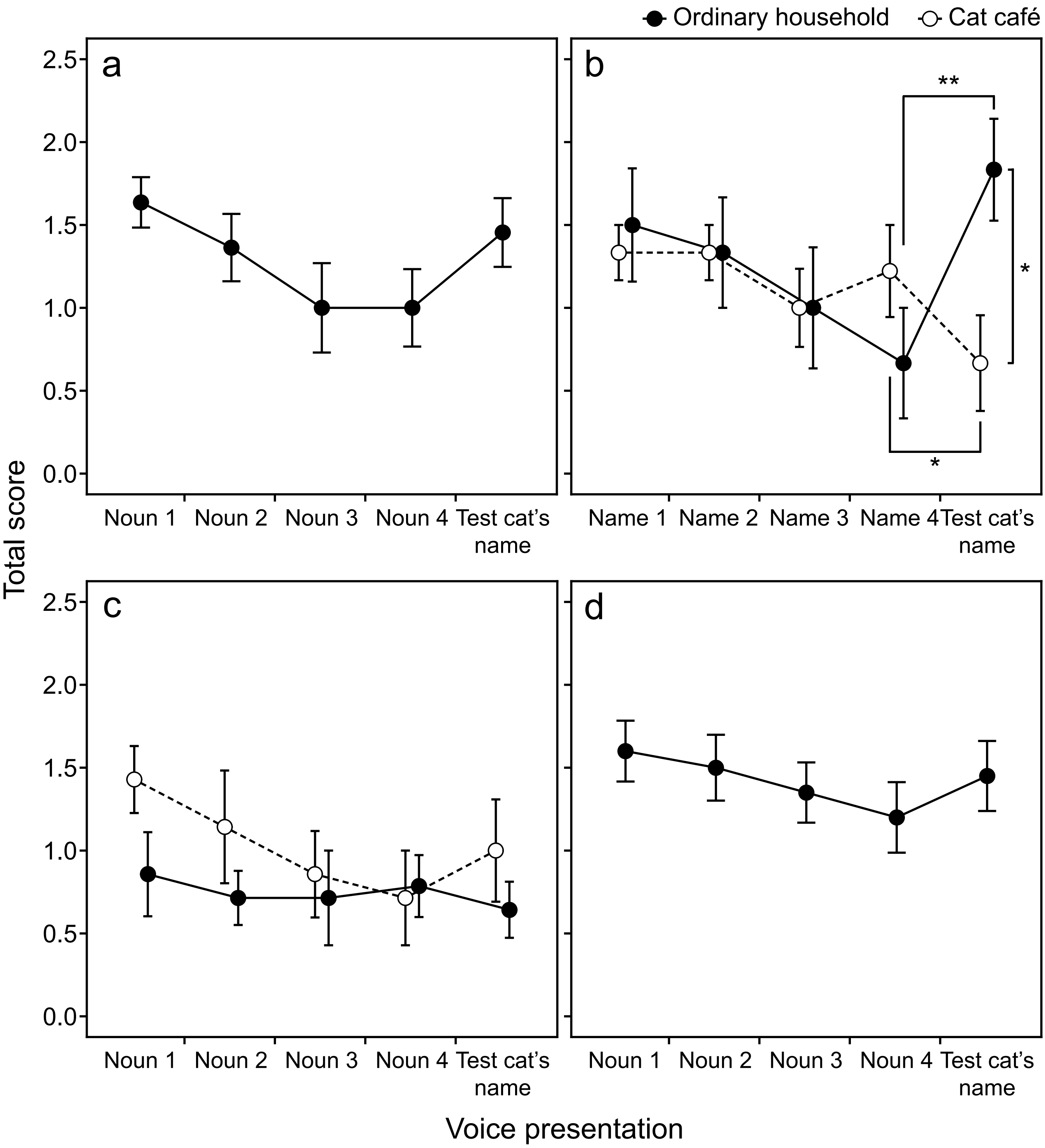

Figure S1. Total behaviour score in habituated cats. a) Experiment 1, b) Experiment 2, c) Experiment 3, and d) Experiment 4. In Experiment 2, significant interaction between trial and environment was observed ($F(1, 13) = 19.67$, $P = 0.001$, GLMM). Significantly increased score in own name was only observed in ordinary household cats ($t(13) = -3.88$, $P = 0.002$). In contrast, café cats significantly decreased score ($t(13) = 2.26$, $P = 0.042$), suggesting continuous habituation from others' name to own name. These two groups were also significantly different in own name trial ($t(19.09) = -2.68$, $P = 0.015$). In Experiment 1, 3, and 4, no significant effect was revealed. Error bars indicate SEs. ** and * indicate $P < 0.01$ and $P < 0.05$ respectively.
